# Optimization of Artificial Intelligence System by Evolutionary Algorithm for Prediction of Axial Capacity of Rectangular Concrete Filled Steel Tubes under Compression

**DOI:** 10.3390/ma13051205

**Published:** 2020-03-07

**Authors:** Hung Quang Nguyen, Hai-Bang Ly, Van Quan Tran, Thuy-Anh Nguyen, Tien-Thinh Le, Binh Thai Pham

**Affiliations:** 1 Thuyloi University, Hanoi 100000, Vietnam; 2 University of Transport Technology, Hanoi 100000, Vietnam; quantv@utt.edu.vn (V.Q.T.); anhnt@utt.edu.vn (T.-A.N.); binhpt@utt.edu.vn (B.T.P.); 3 Institute of Research and Development, Duy Tan University, Da Nang 550000, Vietnam

**Keywords:** axial capacity prediction, rectangular CFST columns, feedforward neural network, invasive weed optimization, hybrid machine learning

## Abstract

Concrete filled steel tubes (CFSTs) show advantageous applications in the field of construction, especially for a high axial load capacity. The challenge in using such structure lies in the selection of many parameters constituting CFST, which necessitates defining complex relationships between the components and the corresponding properties. The axial capacity (P_u_) of CFST is among the most important mechanical properties. In this study, the possibility of using a feedforward neural network (FNN) to predict P_u_ was investigated. Furthermore, an evolutionary optimization algorithm, namely invasive weed optimization (IWO), was used for tuning and optimizing the FNN weights and biases to construct a hybrid FNN–IWO model and improve its prediction performance. The results showed that the FNN–IWO algorithm is an excellent predictor of P_u_, with a value of R^2^ of up to 0.979. The advantage of FNN–IWO was also pointed out with the gains in accuracy of 47.9%, 49.2%, and 6.5% for root mean square error (RMSE), mean absolute error (MAE), and R^2^, respectively, compared with simulation using the single FNN. Finally, the performance in predicting the P_u_ in the function of structural parameters such as depth/width ratio, thickness of steel tube, yield stress of steel, concrete compressive strength, and slenderness ratio was investigated and discussed.

## 1. Introduction

Concrete and steel are the two most commonly used construction materials today. However, each material has different advantages and disadvantages [1,2,3]. Therefore, to be able to take advantages and minimize disadvantages, an optimal solution is to use a combination of both materials, such as a “combined steel concrete structure” or using a combination of concrete elements and steel elements in “composite structures”. One of the combined steel concrete structures is a steel pipe composite structure filled with medium or high strength concrete. This type of structure is called a steel-concrete pipe.

In recent decades, concrete filled steel tubes (CFSTs) have been widely used in the construction of modern buildings and bridges [4], even in high seismic risk areas [5,6,7,8,9,10]. This increase in use is because of the significant advantages that the CFST column system offers over conventional steel or reinforced concrete systems, such as high axial load capacity [4], good plasticity and toughness [6], larger energy absorption capacity [7], convenient construction [11], economy of materials [12,13,14], and excellent seismic and refractory performance [15]. In particular, this type of structure can reduce the environmental burden by removing formwork [16], reusing steel pipes, and using high quality concrete with recycled aggregate [17]. The characteristics of CFST are that the steel material is located far from the central axis so the rigidity of the column is very large, and thus it also contributes to increasing the moment of inertia of the structure [5,18]. The ideal form of concrete core works against the compressive load and hinders the local buckling state of the steel pipe. Therefore, the CFST structures are often used in locations subject to large compressive loads [9,15,19]. The CFST columns are mainly divided into square columns, round columns, and rectangular columns, based on different cross-sectional forms [15]. In particular, the square and rectangular CFST columns have the advantage of easy connection and reliable work with other structural members such as beams, walls, and panels [20]. Compared with square CFST columns, rectangular columns have irregular bending stiffness along different axes, so this type of column is suitable for the mechanical behavior of members including arch ribs, pillars, abutments, and piers, and other structural members under load actions vary greatly from vertical to horizontal [6]. Because the scope of application of rectangular CFST columns is quite wide and this column is mainly subjected to compression, the main purpose of the paper is to analyze and evaluate the ultimate bearing capacity of rectangular columns.

In recent decades, the regulations for calculating the CFST column type have been proposed in design standards such as AISC-LRFD [21], ACI 318-05 [22], Japan Institute of Architecture [17], European Code EC 4, British Standard BS 5400 [23], and Australian Standard AS-5100.6 [24]. In addition, numerous experimental and numerical studies were conducted to analyze the mechanical properties of rectangular CFST columns under axial compression. As an example, Hatzigeorgiou [25] has proposed a theoretical analysis for modeling the behavior of CFST under extreme loading conditions. Later, the verification of such an approach against experimental and analytical results has also been reported in the work of Hatzigeorgiou [26]. In the work of Liu et al. [4], 26 rectangular CFST column samples were experimented under concentric compression with the main parameters such as strength and aspect ratio. In Chitawadagi et al. [8], the load capacity of CFST columns depended on the variation of CFST properties such as the wall thickness of pipes, strength of in-filled concrete, area of cross section of steel pipes, and pipe length. In this study, 243 rectangular CFST samples were investigated; the experimental results were compared with the predicted column strength, which was performed according to design codes such as EC4-1994 and AISC-LRFD-1994. In addition, there are many other test methods dealing with factors that affect the bearing capacity of rectangular CFST columns such as the effect of concrete compaction [27], load conditions, and boundary conditions [16]. The addition of steel fibers in core concrete had a significant effect on the performance of concrete steel pipes [28] and many other tests [9,13,29,30,31,32]. Finite element analysis is now also frequently used for design and research issues thanks to the existence of many commercial software such as ABAQUS [33] and ANSYS [34]. Tort et al. [35] carried out computational research to analyze the nonlinear response of composite frames including rectangular concrete pipe beams and steel frames subjected to static and dynamic loads. On the basis of the Drucker–Prager model, Wang et al. [36] developed a finite element model that can predict the axial compression behavior of a composite column with a fibrous reinforced concrete core. Collecting 340 test data of circular, square, and rectangular CFST columns, Tao et al. [37] developed new finite element models for simulating CFST stub columns under compression mode along the axis. The new model was more flexible and accurate for modeling the CFST stub columns. However, the design standards were limited by the scope of use and were not suitable for high-strength materials, and testing methods were often costly and time-consuming. The accuracy of finite element models was greatly affected by the input parameters, especially the suitable selection of the concrete model. Therefore, it is necessary to propose a uniform and effective approach to design rectangular CFST columns.

In recent years, artificial intelligence (AI) based on computer science has gradually become popular and applied in many different fields [38,39,40,41]. Artificial neural network (ANN) is a branch of AI techniques; different ANN-based modeling methods have been used by scientists in many construction engineering applications [42]. Sanad et al. [43] used ANN to estimate the reinforced concrete deep beams ultimate shear strength. Lima et al. [44] predicted the bending resistance and initial stiffness of steel beam connection using a back-propagation algorithm. Seleemah et al. [45] applied ANN to predict the maximum shear strength of concrete beams without horizontal reinforcement. Blachowski and Pnevmatikos [46] have developed a vibration control system based on the ANN method, for application in earthquake engineering. As an example for structural engineering, Kiani et al. [47] have applied AI techniques including support vector machines (SVM) and ANN for deriving seismic fragility curves. It is worth noticing that significant studies have been carried out to explore the prediction of damage using AI techniques. In a series of papers, Mangalathu et al. [48] have proposed various AI methods such as ANN and random forest for tracking damage of bridge portfolios [48] as well as assessing the seismic risk of skewed bridges [49]. In terms of structural failure, typical failure modes of reinforced concrete columns such as flexure, flexure–shear, and shear were investigated by Mangalathu et al. [50,51] using decision trees (DT), SVM, and ANN. Guo et al. [52,53] employed the ANN model for the identification of damage in different structures such as suspended-dome and offshore jacket platforms. Regarding structural uncertainty analysis, various published works by E. Zio should be consulted [54,55,56]. With rectangular CFST columns, the use of ANN has also been proposed. For example, Sadoon et al. [57] proposed an ANN model for predicting the final strength of rectangular concrete steel beam girder (RCFST) under eccentric shaft load. The results showed that the ANN model was more accurate than the AISC and Eurocode 4 standard. Du et al. [10] formulated an ANN model with different input parameters to determine the axial bearing capacity of rectangular CFST column. The results of the model were compared with the results calculated according to European Code EC 4 [23], ACI [22], and AISC360-10 [21], and found that the ANN model was accurate. However, in the above studies, the mentioned correlation coefficient (R) was less than 0.98. Therefore, in this paper, we tried to create a bulk sample set and proposed an algorithm to increase the accuracy of the prediction of the axial load bearing capacity of the CFST column.

In short, the aim of this paper is dedicated to the development and optimization of an AI-based model, namely the feedforward neural network (FNN), to predict the P_u_ of CFST. An optimization algorithm, invasive weed optimization (IWO), was used to finely tune the FNN parameters (i.e., weights and biases) to develop a hybrid model, namely FNN–IWO, and to improve the prediction performance. With respect to the CFST database, 99 samples were collected from the available literature and used for the training and testing phases of the FNN–IWO algorithm. Criteria such as coefficient of determination (R^2^), standard deviation error (ErrorStD), root mean square error (RMSE), mean absolute error (MAE), and slope were used to evaluate the performance of FNN–IWO. Finally, an investigation of the prediction capability in the function of different structural parameters was conducted.

## 2. Materials and Methods

### 2.1. Feedforward Neural Network (FNN)

An artificial or neural network (also known as an artificial neural network (ANN)) is a biological neural network based a computational or mathematical model. It includes a number of artificial neurons (nodes) that are linked to each other and processes information by transmitting along the connections and calculating new values at the nodes (connection method for calculation) [58,59]. The ANN models are made up of three or more layers, including an input layer that is the leftmost layer of the network representing the inputs, an output layer that is the rightmost layer of the network representing the results achieved, and one or more hidden layers representing the logical reasoning of the network [60,61,62]. The neurons in each layer are linked to the front and rear neurons with each associated weight. A training algorithm is often used to repeat minimizing the cost function relative to the link weight and neuron threshold. Networks are usually divided into two categories based on how the units are connected, including the feedforward neural network (FNN) and the recurrent neural network. To date, FNN is the most popular architecture owing to its structural flexibility, good performance, and the availability of many training algorithms [63]. Currently, the most widely used training algorithm for multi-layer feedforward networks is the backpropagation algorithm (BP). In BP, network training is achieved by adjusting weights and is done through numerous training sets and training cycles [64]. With the ability to approximate the functions, FNNs have been successfully applied in a number of civil engineering and structural fields [65] such as predicting the compression strength of concrete [66], investigating the fire resistance of calves [67], determining the axial strength of cylindrical concrete pillars [58], and predicting the fire resistance of concrete tubular steel columns [65]. Therefore, in this study, FNN was selected and used to predict the axial capacity of CFST.

### 2.2. Invasive Weed Optimization (IWO)

IWO is a new random number optimization method inspired by a popular phenomenon in agriculture. The term of weed invasion was first introduced by Mehrabian and Lucas in 2006 [68]. This technique is based on a number of interesting features of invasive weed plants that reproduce and distribute fast and vigorously, and adapt themselves to changes in climatic conditions [69]. Therefore, capturing their characteristics will lead to a powerful optimization algorithm [70]. The advantages of IWO algorithm compared with other evolutionary algorithms are few parameters, simple structure, easy to understand, and easy to program features [71]. Up to now, the IWO algorithm has become more and more popular and has been successfully applied in areas such as antenna system design [72] and design of coding chains for DNA [73], as well as inter-related problems regarding economic [74], tourism [75], and construction techniques [76]. The IWO algorithm is implemented by the following steps:

Step 1. Initialization: Weeds are randomly scattered over a D-dimensional target area as the primary solution.
Step 2. Reproduction: During reproduction, each weed produces seed depending on the physical strength and colony. Weeds that acquire more resources have a better chance of producing seeds and plants that are less adapted to fields are not able to reproduce, and thus produce fewer seeds. The number of seeds increases linearly from the minimum value for the worst weed to the maximum value for the best weed.
Step 3. Spatial dispersal: The seeds generated from step 2 are randomly dispersed in the search space by means of normally distributed random numbers with an average of zero, but with different variances to ensure that the seeds are located around the main factory.
Step 4. Competitive exclusion: The spawning and dispersal process randomly create a new population for the next generation of weeds and their seeds. When the size of this new population is greater than a certain maximum value, the lower-strength weeds will be eliminated through competition and only some of the weeds will be equal to the dark weed population.
Step 5. Termination conditions: The process continues again from step 2 to step 4 until the maximum number of iterations is reached and the best physical tree is nearest to the optimized solution.

### 2.3. Quality Assessment Criteria

Evaluation of the AI model was performed using statistical measurements such as mean absolute error (MAE), coefficient of determination (R^2^), and root mean square error (RMSE). In general, these criteria are popular methods to quantify the performance of AI algorithms [76,77]. More specifically, the mean squared difference between actual values and estimated values defines RMSE, whereas the mean magnitude of the errors defines MAE. The R^2^ evaluates the correlation between actual and estimated values [78,79,80]. Quantitatively, lower RMSE and MAE show better performance of the models. In contrast, a higher R^2^ shows better performance of the model [81,82]. MAE, RMSE, and R^2^ are expressed as follows [83,84]:

(1) $$\mathrm{MAE}=\frac{1}{N}\sum_{i=1}^{N}\left(a_{i}-{\bar{a}}_{i}\right)$$

(2) $$\mathrm{RMSE}\;=\;\sqrt{\frac{1}{N}\sum_{i=1}^{N}{\left(a_{i}-{\bar{a}}_{i}\right)}^{2}}$$

(3) $$R^{2}=1-\frac{\sum_{i=1}^{N}{\left(a_{i}-{\bar{a}}_{i}\right)}^{2}}{\sum_{i=1}^{N}{\left(a_{i}-\bar{a}\right)}^{2}}$$

where $a_{i}$ is the actual output, ${\bar{a}}_{i}$ infers the predicted output, $\bar{a}$ infers the mean of the $a_{i}$, and *N* infers the number of used samples.

### 2.4. Data Used and Selection of Variables

**Figure 1:**
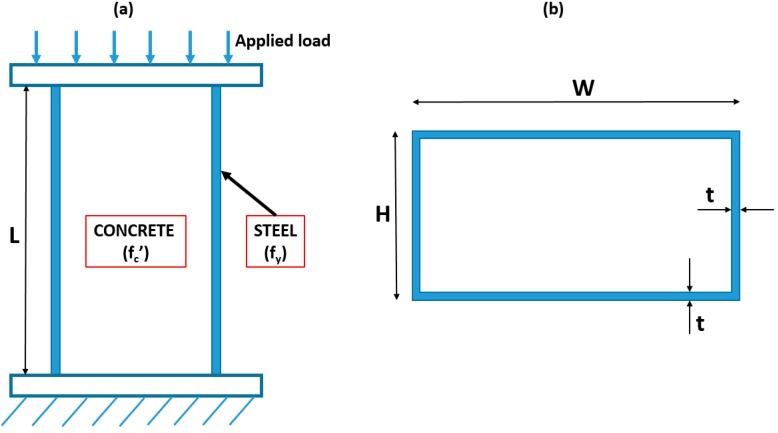
Schematic diagram of the compression test for concrete filled steel tubes (CFSTs): (**a**) front view; (**b**) cross-section view of the sample.

In this study, a total of 99 compression tests of rectangular CFST columns (Figure 1) were extracted from the available literature: Bridge [85], Du et al. [86], Du et al. [87], Ghannam et al. [88], Han [89], Han & Yang [90], Han & Yao [91], Lin [92], Schneider [93], Shakir-Khalil & Mouli [94], and Shakir-Khalil & Zeghiche [95]. Information of the database is summarized in Table 1, including the number of data and the percentage of proportion, whereas Table 2 presents the initial statistical analysis of the corresponding database.

The experimental tests were carried out considering the following steps: design, processing of steel tube, production of concrete, curing of specimens, and loading measurement [15,86]. As proposed by Sarir et al. [96] and Ren et al. [15] in investigating CFST columns, initial geometric imperfections as well as residual stress exhibited a negligible effect on the behavior of columns under axial loading. Consequently, input variables affecting the axial capacity of rectangular CFST are from two main groups: geometry of columns and mechanical properties of constituent materials. Therefore, six independent variables were selected as inputs of the problem, such as depth of cross section (H), width of cross section (W), thickness of steel tube (t), length of column (L), yield stress of steel (f_y_), and compressive strength of concrete (f_c_’). It is seen in Table 2 of the initial statistical analysis that all input variables cover a wide range of values. More precisely, H varies from 90 to 360 mm with an average value of 163 mm and a coefficient of variation of 32%. W ranges from 60 to 240 mm with an average value of 111 mm and a coefficient of variation of 32%. t ranges from 0.7 to 10 mm with an average value of 4 mm and a coefficient of variation of 48%. L varies from 100 to 3050 mm with an average value of 869 mm and a coefficient of variation of 89%. f_y_ ranges from 194 to 515 MPa with an average value of 329 MPa and a coefficient of variation of 24%. f_c_’ varies from 8 to 47 MPa with an average value of 31 MPa and a coefficient of variation of 39%.

**Figure 2:**
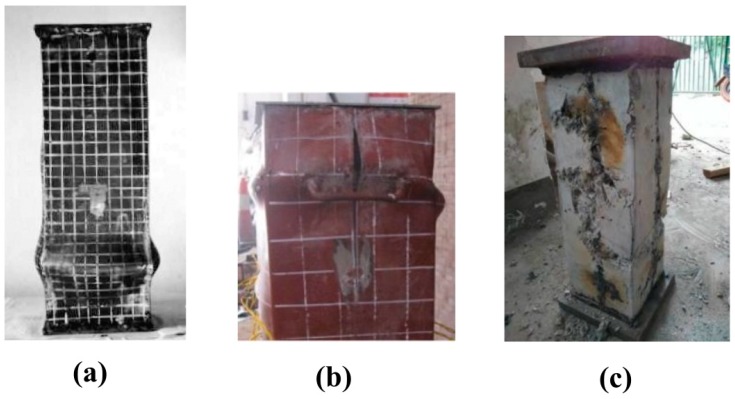
Failure of rectangular CFST specimens: (**a**) local outward buckling of steel tube (reproduced with permission from Han [89]), (**b**) tensile fracture at the welding seam of steel tube (reproduced with permission from Ding et al. [98]), (**c**) damage of concrete core (reproduced with permission from Lyu et al. [97]).

It should be pointed out that the steel tube of 43 specimens was cold-formed, whereas welded built-up was done in the other 56 configurations. In terms of failure modality, local outward buckling failure of the external steel was observed in all specimens, as shown in Figure 2a. This is the same as that observed by other investigations such as Han and Yao [91], Lyu et al. [97], Ding et al. [98], and Yan et al. [99]. Depending on the dimension of the cross section, the locations of the external folding of the steel tube are not the same. Such local buckling of the steel tube occurred mostly at the ends or in the center along the axis of the specimens, as seen in Figure 2a. In addition to outward buckling failure, fracture at the welding seam also occurred in welded specimens, as shown in Figure 2b. Such tensile fracture is the result of too much growth of the concrete in the core [99]. However, the tensile fracture of the steel tube generally occurred after the peak load [98]. Last, but not least, for all specimens, concrete in the core was damaged in most of specimens following a shear failure mode, as shown in Figure 2c [97,98]. Besides, the influence of temperature on the failure modality of stub CFST structural members could be referred to in Yan et al. [99] (low temperature) and Lyu et al. [97] (high temperature). Finally, Angelo et al. [100] and Kulkarni et al. [101] have tested and discussed about the failure of rectangular CFST structural members in junction with wide beam for earthquake engineering application.

It is worth mentioning that only rectangular CFST columns (i.e., depth/width ratio greater than 1) were collected for investigation. As indicated in Table 2, the depth/width ratio ranges from 1 to 2, allowing for exploring the axial failure of CFST around the weak axis. In addition, as the depth/width ratio differs than 1, the stress of confined concrete applied to the steel wall is not the same along the weak and strong axes, while the thickness of the steel tube was constant. Consequently, the consideration of only rectangular CFST columns could strongly reveal the influence of both the structural geometry and mechanical properties of constituent materials.

**Figure 3:**
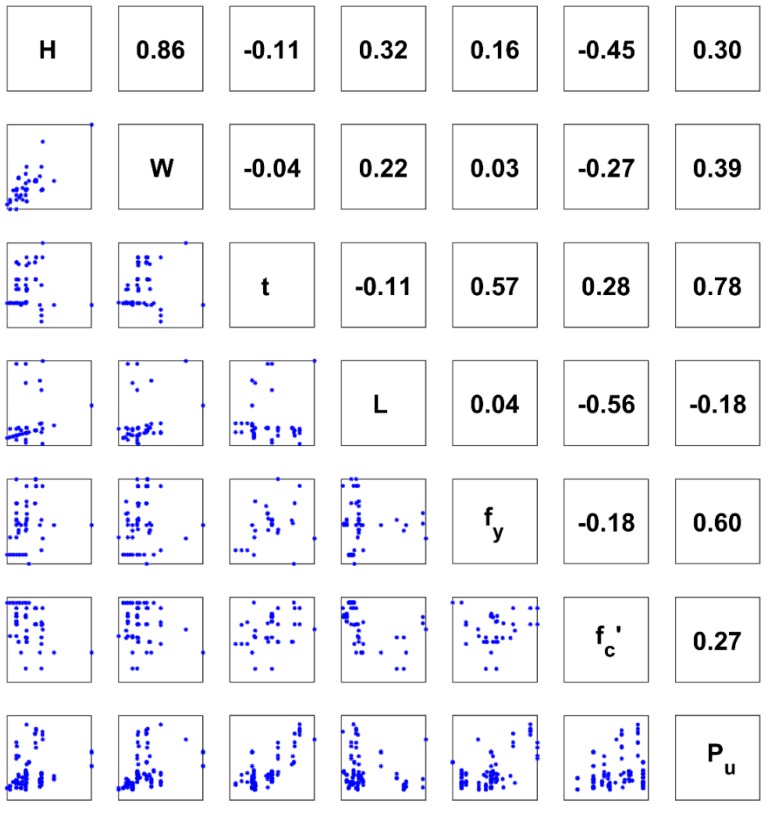
Correlation analysis between the depth of cross section (H), width of cross section (W), thickness of steel tube (t), length of column (L), yield stress of steel (f_y_), concrete compressive strength (f_c_’), and axial capacity (P_u_).

The dataset was randomly divided into two sub-datasets including the training part (60%) and testing part (40%) part. All data were scaled into the range of [0,1] in order to reduce numerical biases while treating with the AI algorithms, as recommended by various studies in the literature [102,103,104]. Such a scaling process is expressed using Equation (4) between raw and scaled data [105,106,107]:

(4) $$x^{scaled}=\frac{\left(x^{raw}-\beta \right)}{\alpha -\beta }$$

where *α* and *β* are the maximum and minimum values of the considered variable *x*, respectively. It should be noticed that a reverse transformation could be used for converting data from the scaling space to the raw one using Equation (4). Besides, a correlation analysis between the input and output variables is performed and plotted in Figure 3.

Figure 3 was generated in order to explore the linear statistical correlation between variables in the database. Therefore, a 7 × 7 matrix was generated, in which the upper triangular part indicates the value of the correlation coefficient, whereas the lower triangular part shows the scatter plot between two associated variables. The diagonal of the matrix indicates the name of the variable (i.e., as the correlation coefficient of a variable itself is equal to 1). For interpretation purpose, the correlation coefficient between H and W is indicated as 0.86, whereas the corresponding scatter plot between H and W is shown on the left side of W (row 2, column 1). It is seen that a high and positive value of statistical correlation was obtained in this case, confirmed by most of the data points being located around the diagonal in the scatter plot.

It can be seen that no direct correlation was observed between each input and output (P_u_). The maximum value of the Pearson correlation coefficient (r) compared with P_u_ was calculated as 0.78 (for variable t), followed by 0.60 (for variable f_y_), 0.39 (for variable W), 0.30 (for variable H), 0.27 (for variable f_c_’), and 0.18 (for variable L). Besides, the correlation between H and W was highest (r = 0.86).

## 3. Results and Discussion

### 3.1. Optimization of Weight Parameters of FNN using the IWO Technique

In this section, the optimization of weight parameters of FNN is presented using the IWO algorithm. It is not worth noticing that the architecture of the FNN model is very important. Depending on the problem of interest, the prediction results could exhibit significant variation from using one architecture to another [96,107,108]. As the numbers of inputs and outputs are fixed, the undetermined parameters of the architecture are the number of hidden layer(s) and the number of neurons in each hidden layer(s) [109]. As proved by many investigations in the literature, the FNN model involving only one hidden layer could be sufficient for exploring successfully complex nonlinear relationship between inputs and outputs. For instance, Mohamad et al. [110] have used one hidden layer architecture model for predicting ripping production, as have Singh et al. [111] for predicting cadmium removal. In civil engineering application, a prediction model involving one hidden layer has also been widely applied in many works, for instance, Gordan et al. [112] for earthquake slope stability or Sarir et al. [96] for bearing capacity of circular concrete-filled steel tube columns. Therefore, the one hidden layer FNN model was finally adopted in this work, also saving cost, processing time, and limitation of instruments. On the other hand, the number of neurons in the hidden layer was recommended to be equal to the sum of the number of inputs and outputs [109,113,114]. Consequently, the FNN model exhibits one hidden layer and seven neurons in the hidden layer. The activation function for the hidden layer was chosen as a sigmoid function, whereas the activation function for the output layer was a linear function [115]. The cost function was chosen such as the mean square error function [116]. Finally, Table 3 indicates the information of the FNN model.

As revealed in the literature, a key aspect of using evolutionary algorithms for optimizing AI models is to study the relationship between population size and problem dimensionality [117,118,119,120]. In many other evolutionary algorithms such as differential evolution, the number in the population is recommended to be 7–10 times the number of inputs [121,122]. In this study, the population size of the IWO technique was chosen as 50. Other parameters include the variance reduction exponent, chosen as 2; initial value of standard deviation, chosen as 0.01; final value of standard deviation, chosen as 0.001; and maximum iteration, chosen as 800. It is worth noticing that such ranges of parameters are commonly employed for training AI models using IWO algorithm, for instance, Huang et al. [76] and Mishagi et al. [123]. It should also be noticed that a large population size cannot be useful in evolutionary algorithms and affects the optimization results [124]. Information of the IWO algorithm is presented in Table 3.

**Figure 4:**
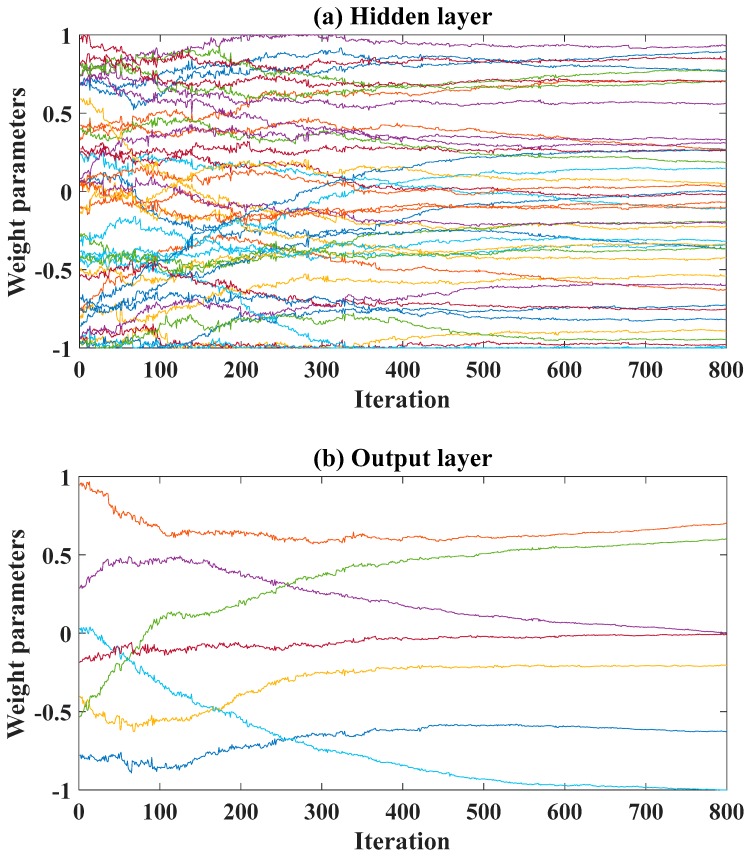
Evolution of weight parameters over 800 iterations: (**a**) weight parameters of input layer (42 parameters); (**b**) weight parameters of hidden layer (7 parameters).

Figure 4a presents the evolution of 42 weight parameters of the hidden layer, whereas Figure 4b shows such evolution of 7 weight parameters of the output layer. It is seen that, at the 300 first iterations, fluctuations were observed for all weight parameters, as the IWO algorithm imitated the colonizing behavior of weed plants. After about 500–600 iterations, stabilization was achieved for weight parameters for the 57-dimensional optimization problem. Consequently, at least 700–800 iterations are needed in order to ensure the stabilization of the process.

**Figure 5:**
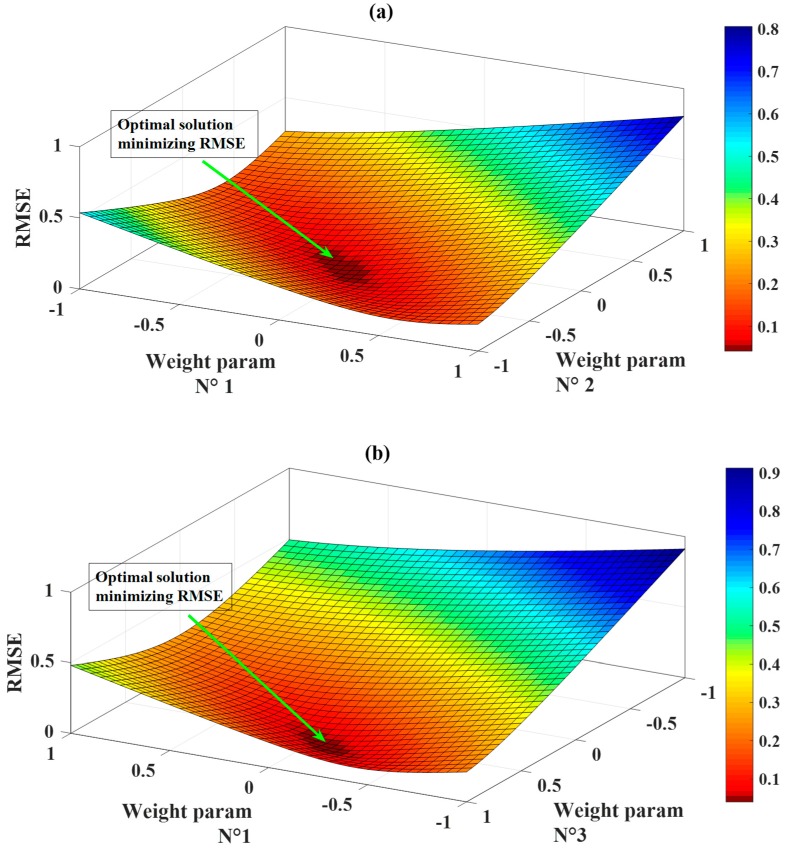
Verification of global optimum provided by the invasive weed optimization (IWO). The surfaces of root mean square error (RMSE) show unique optimal solution, which minimizes the value of RMSE: (**a**) between weight parameters N°1 and N°2, (**b**) between weight parameters N°1 and N°3.

Weight parameters at iteration 800 were extracted for constructing the final FNN–IWO model (a combination of FNN and IWO). This model was then used as a numerical prediction function for parametrically investigating the deviation of quality assessment criteria in function weight parameters. The parametric study could be helpful to verify if the results provided by the IWO were unique, that is, the IWO allowed reaching the global optimum of the problem. For illustration purposes, only three first weight parameters were plotted. Figure 5a presents the evolution of RMSE while varying weight parameters N°1 and N°2 from their lowest to highest values. In the same context, Figure 5b presents the evolution of RMSE while varying weight parameters N°1 and N°3 from their lowest to highest values. It is seen from Figure 5a,b that the global optimum of the two RMSE surfaces matched the final set of weight parameters provided by the IWO algorithm. This remark confirmed that the IWO technique allowed calibrating the global optimum of the optimization problem, thus providing the final FNN–IWO model.

**Figure 6:**
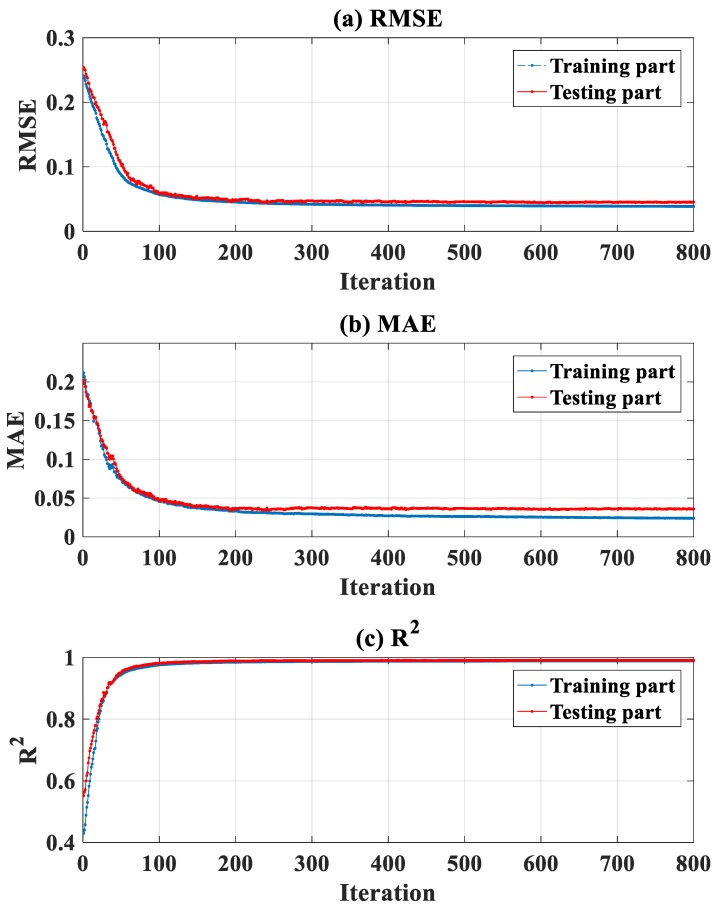
Evaluation of the performance indicators during optimization: (**a**) RMSE, (**b**) mean absolute error (MAE), and (**c**) R^2^, for training and testing data, respectively.

Figure 6a–c present the evolution of RMSE, MAE, and R^2^ during the optimization process of FNN weight parameters, for both training and testing data. It is seen that during the optimization using the training data, good results of RMSE, MAE, and R^2^ for the testing data were obtained. It is not worth noting that the testing data were totally new when applying. This remark allows exploring that no overfitting occurred during the training phase (i.e., performance indicators of testing data go in a bad direction). The efficiency and robustness of the IWO technique are then confirmed.

### 3.2. Influence of the Training Set Size

**Figure 7:**
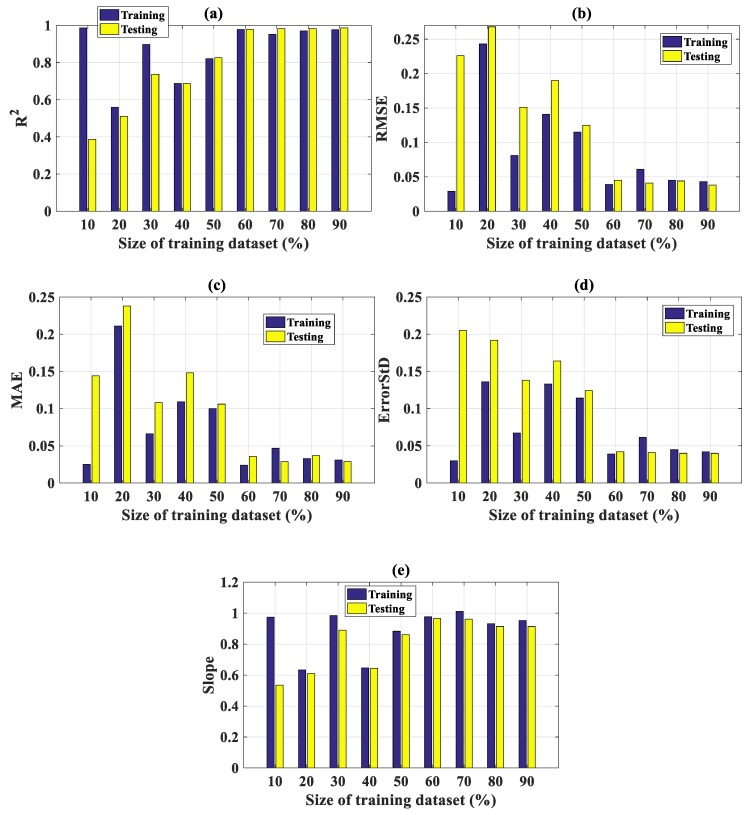
Influence of training set size with respect to (**a**) R^2^, (**b**) RMSE, (**c**) MAE, (**d**) ErrorStD, and (**e**) slope.

In this section, the influence of training set size (in %) on the prediction results is presented. The training dataset was varied from 10% to 90% of the total data (with a resolution of 10%). Figure 7 illustrates the influence of training set size, with respect to R^2^ (Figure 7a), RMSE (Figure 7b), MAE (Figure 7c), ErrorStD (Figure 7d), and slope (Figure 7e). All relevant values are also highlighted in Table 4.

As seen in Figure 7a,e for R^2^ and slope, the performance of the prediction model progressively increased during the increasing of the training set size from 10% to 90%. For instance, for the testing part, R^2^ = 0.387 when the training set size was 10%, which was increased to 0.987 when the training set size was 90%. The same remark was also obtained when regarding Figure 7b,c,d for RMSE, MAE, and ErrorStD, respectively. Moreover, the performance of the prediction model for both training and testing parts became stable from 60% of the training set size (Figure 7a). This observation indicates that no over-fitting occurred when the training set size surpassed a high percentage, for instance, 80%. This point proves that the prediction model is robust, exhibiting a strong capability in tracking relevant information in the testing part even it is small. Finally, yet importantly, the prediction model is promising in the case in which more data are available.

### 3.3. Prediction Capability of the FNN–IWO Model

**Figure 8:**
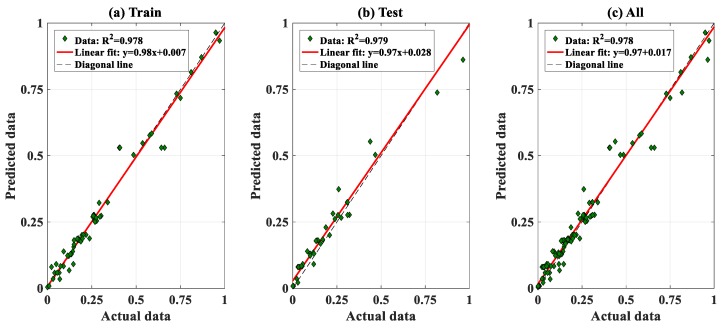
Comparison between actual and predicted data in regression scatter mode for (**a**) training data, (**b**) testing data, and (**c**) all data.

In this section, the performance of FNN–IWO in predicting the P_u_ of CFST is investigated. The predicted outputs versus the corresponding experimental results associated with the training, testing, and all datasets are presented in Figure 8. The fitted linear lines are also plotted (red lines) in each graph to show the performance of the algorithm. R^2^ values with respect to the training, testing, and all datasets were estimated at 0.978, 0.979, and 0.978, respectively, showing an excellent prediction capability of FNN–IWO. Furthermore, three linear equations representing the relationships between actual and predicted data were also given in each graph, including the intercepts and slopes. It is observed that the FNN–IWO algorithm possessed a strong linear correlation between actual and predicted P_u_ values.

The detailed performance of the proposed FNN–IWO algorithm is summarized in Table 5, including R^2^, RMSE, MAE, standard deviation error (ErrorStD), slope, and slope angle. Regarding the results of quality assessment and error analysis, FNN–IWO exhibited a strong capability in predicting the critical compression capacity of the rectangular section.

**Figure 9:**
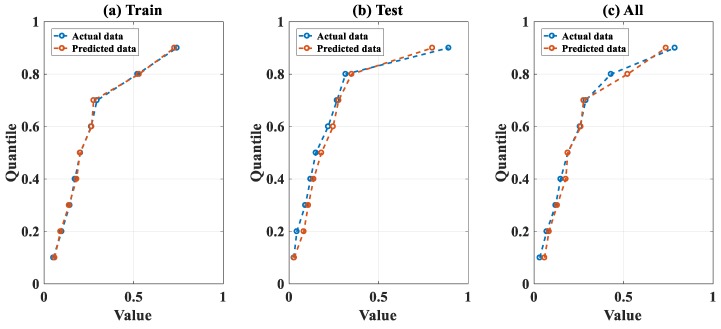
Comparison between actual and predicted data at different quantile levels of the distributions for (**a**) training data, (**b**) testing data, and (**c**) all data.

**Figure 10:**
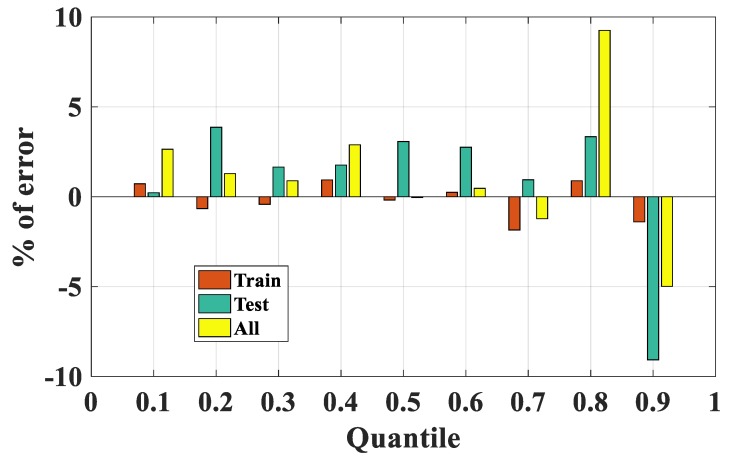
Percentage of error between quantile estimation for training, testing, and all data.

For further assessment of the performance of the FNN–IWO algorithm, comparison between the experimental and predicted results was performed at different quantile levels. For this purpose, quantiles from 10% to 90% were computed to track the behavior of the distribution of the data, with a focus on the most important statistical distribution. The results are presented (Figure 9a–c) for the training, testing, and all data, respectively, whereas the percentage of error (%) between the predicted and actual values at each quantile level is displayed in Figure 10.

It is seen that, for the training dataset, the actual and predicted data were highly correlated, whereas a small difference was observed at each level of quantile for the testing part. With respect to the whole dataset, the highest error ratio was observed at Q80, followed by Q90 and Q10. For the values of error, it was seen that the FNN–IWO model exhibited a strong efficiency in predicting P_u_ within the Q10–Q70 range (error < 5%) and from Q80 to Q90 (with error in the 5%–10% range).

### 3.4. Prediction Accuracy in Function of Structural Parameters of FNN–IWO

**Figure 11:**
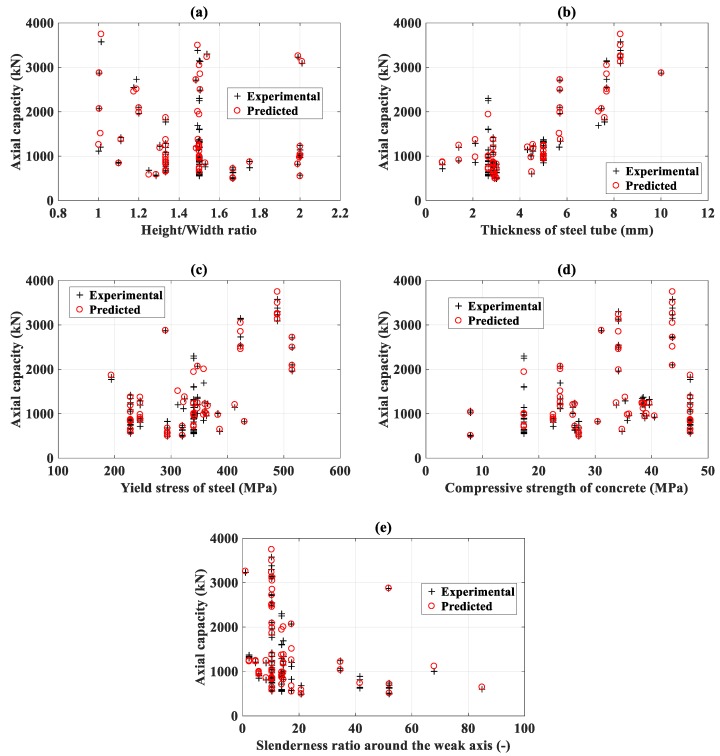
Evaluation of axial capacity in function of the (**a**) depth/width ratio, (**b**) thickness, (**c**) yield stress, (**d**) compressive strength, and (**e**) slenderness ratio.

In this section, the prediction accuracy of FNN–IWO with respect to different ranges of structural parameters is presented. The actual and predicted P_u_ in function of the depth /width ratio, t, f_y_, f_c_’, and slenderness ratio are displayed in Figure 11a–e, respectively. Besides, error analysis in terms of R^2^, RMSE, and MAE for several intervals of the depth/width ratio, t, f_y_, f_c_’, and slenderness ratio, respectively, is also indicated in Table 6 and Figure 11, together with the associated number of data.

In the case of the depth/width ratio, 11 configurations were found between 1 and 1.2, exhibiting R^2^ = 0.98, RMSE = 137.57 kN, and MAE = 95.25 kN; 22 configurations were found between 1.2 and 1.4, showing R^2^ = 0.98, RMSE = 71.07 kN, and MAE = 56.01 kN; 43 configurations were found between 1.4 and 1.6, exhibiting R^2^ = 0.97, RMSE = 144.71 kN, and MAE = 109.65 kN; 11 configurations were found between 1.6 and 1.8, exhibiting R^2^ = 0.89, RMSE = 56.16 kN, and MAE = 38.91 kN; and only 3 configurations were found between 1.8 and 2, exhibiting R^2^ = 1.00, RMSE = 24.75 kN, and MAE = 21.81 kN. Such an analysis allowed confirming that the FNN–IWO model is efficient in predicting P_u_ from nearly square to highly rectangular columns.

In the case of slenderness, 78 configurations were found between 0 and 20 of slenderness, exhibiting R^2^ = 0.98, RMSE = 123.29 kN, and MAE = 86.64 kN; 6 configurations were found between 20 and 40 of slenderness, showing R^2^ = 0.98, RMSE = 42.80 kN, and MAE = 32.09 kN; 13 configurations were found between 40 and 60 of slenderness, exhibiting R^2^ = 0.99, RMSE = 72.25 kN, and MAE = 55.32 kN. Although the number of data is small for large slenderness, such an analysis allowed remarking that the FNN–IWO model is efficient in predicting P_u_ for short, medium, and long columns.

### 3.5. Comparison of the Hybrid Model of FNN–IWO and the Single FNN Model

In order to highlight the efficiency of the evolutionary IWO algorithm, comparisons between FNN–IWO and the individual FNN were performed, using a similar training algorithm (scaled conjugate gradient (SCG)), FNN architecture, and dataset.

**Figure 12:**
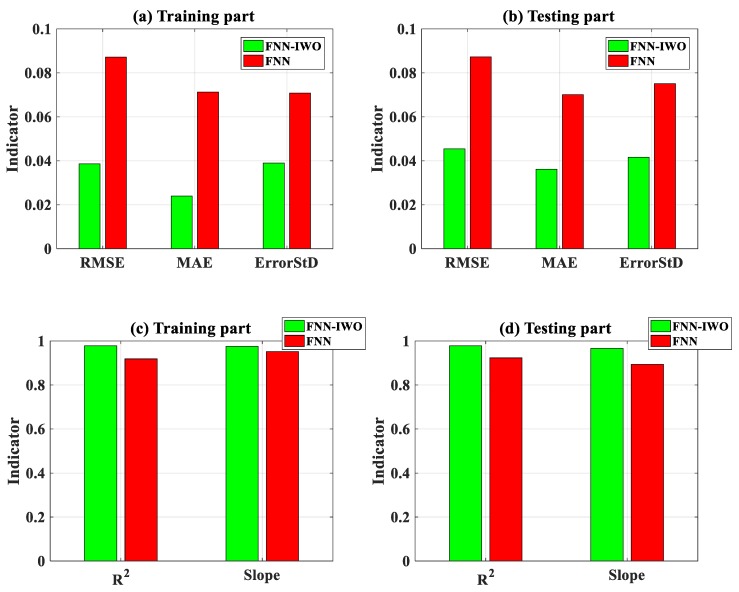
Comparison of performance indicators between the individual FNN and FNN–IWO model: (**a**) RMSE, MAE, and ErrorStD for training data; (**b**) RMSE, MAE, and ErrorStD for testing data; (**c**) R^2^ and slope for training data; and (**d**) R^2^ and slope for testing data.

Considering RMSE, MAE, and standard deviation error (ErrorStD), Figure 12 identifies the values of the two algorithms for the training part (Figure 12a) and testing part (Figure 12b). It can be clearly seen that FNN–IWO is more accurate than the single FNN, represented by a reduction of error for RMSE (2 times), MAE (3 times), or ErrorStD (2 times). Improvement of the accuracy is more pronounced in the training part than the testing part. Considering R^2^ and slope as error criteria, FNN–IWO also exhibited an advantage compared with FNN without optimization, for both the training and testing datasets (Figure 11c,d).

For the sake of comparison, Table 7 indicates the exact values and gains (in %) while using FNN–IWO with FNN for five error criteria. With a focus on the testing part, the gains reached 47.9%, 49.2%, 41.3%, 6.5%, and 1.5% for RMSE, MAE, ErrorStD, R^2^, and slope, respectively. As a conclusion, using IWO to tune the weights and bias of FNN strongly enhanced the accuracy in predicting P_u_.

## 4. Conclusions and Outlook

Even though many studies attempted to predict the P_u_ of CFST with different AI algorithms, the accuracy and robustness of these algorithms still need further comprehensive investigation. In this study, a novel hybrid approach of FNN–IWO was proposed and improved for the prediction of P_u_ of CFST, of which IWO was used for tuning and optimizing the FNN weights and biases to improve the prediction performance.

The results showed that the FNN–IWO algorithm is an excellent predictor of P_u_, with a value of R^2^ of up to 0.979. The performance of FNN–IWO in predicting P_u_ function of structural parameters such as depth/width ratio, thickness of steel tube, yield stress of steel, concrete compressive strength, and slenderness ratio was investigated and the results showed that FNN–IWO is efficient in predicting P_u_ from nearly square to highly rectangular columns, as well as for short, medium, and long columns. Better performance of FNN–IWO was also pointed out with the gains in accuracy of 47.9%, 49.2%, and 6.5% for RMSE, MAE, and R^2^, respectively, compared with the simulation using the single FNN. This study may help in quick and accurate prediction of P_u_ of CFST for better practice purposes.

In general, the main advantage of AI-based methods is its efficient capability to model the macroscopic mechanical behavior of the structural members without any prior assumptions or constraints. Therefore, the developed AI model in this study could be applied to the pre-design phase of the design process. Indeed, such quick numerical estimation is helpful to explore some initial evaluations of the outcome before conducting any extensive laboratory experiments. To this aim, a graphical user interface application should be compiled for facilitating the application by engineers/researchers.

On the other hand, empirical formulae should be derived based on the “black-box” AI-based model developed in this study for estimating the axial behavior of rectangular CFST columns. In addition, the performance of such empirical formulae should be compared with other existing equations in the literature such as Ding et al. [98], Wang et al. [125], and Han et al. [126]. Besides, numerical finite element scheme should also be studied, especially for investigating the mechanical behaviors of composite columns at both the micro and macro levels. Finally, improvement for current designs (such as Eurocode-4 [127], AISC [128], and ACI [129]), if it exists, should be proposed.

The axial behavior of CFST composite columns is a complex problem, involving various variables such as geometry and mechanical properties of constituent materials. Consequently, experimental databases are crucial for studying this problem. In further studies, a larger database should be considered, in order to cover more material strengths and geometric dimension ranges.

The methodology modeling of this work could be extended for predicting other macroscopic properties such as bending, compression, or tension strength of not only composite members, but also members made of a single material (i.e., concrete or steel members). Besides, an investigation based on homogenization and de-homogenization approaches [130,131,132,133,134] could also be useful for studying structural members under different boundary conditions and loadings. Such a framework, including the finite element scheme, could also be coupled with AI-based prediction in order to better understand the micro and macro behaviors of structural members.

## Figures and Tables

**Table 1:** Information of the database used in this study.

No.	Reference	Number of Data	% of Proportion
1	Bridge [85]	1	1.0
2	Du et al. [86]	5	5.1
3	Du et al. [87]	8	8.1
4	Ghannam et al. [88]	12	12.1
5	Han [89]	20	20.2
6	Han & Yang [90]	4	4.0
7	Han & Yao [91]	19	19.2
8	Lin [92]	6	6.1
9	Schneider [93]	9	9.1
10	Shakir-Khalil & Mouli [94]	14	14.1
11	Shakir-Khalil & Zeghiche [95]	1	1.0
	Total	99	100

**Table 2:** Initial statistical analysis of database.

Parameters	Symbol	Unit	Role	Min	Q_25_	Median	Q_75_	Max	Mean	StD	Coefficient of Variation (%)
Depth of cross section	H	mm	Input	90	127.9	150	195	360	163.38	53.01	32.45
Width of cross section	W	mm	Input	60	90	100	124.48	240	110.94	35.63	32.12
Thickness of steel tube	t	mm	Input	0.7	2.7	3	5	10.01	4.12	1.97	47.84
Length of column	L	mm	Input	100	369.75	545	800	3050	869.23	772.12	88.83
Yield stress of steel	f_y_	MPa	Input	194	245.18	340.1	357.88	514.53	329.09	78.73	23.92
Compressive strength of concrete	f_c_’	MPa	Input	7.9	18.67	33.74	43.69	46.85	31.12	12.21	39.23
Axial capacity	P_u_	kN	Output	490	760	1006	1340	3575	1267.61	768.72	60.64

**Table 3:** Values and description of feedforward neural network (FNN) and invasive weed optimization (IWO) parameters in this study.

Methods	Parameter	Values and Description
FNN	Number of neurons in input layer	6
	Number of neurons in output layer	1
	Number of hidden layers	1
	Number of neurons in hidden layer	7
	Size of weight matrix of hidden layer	42
	Size of weight matrix of output layer	7
	Size of bias vector of hidden layer	7
	Size of bias vector of output layer	1
	Dimension of optimization problem	57
	Activation function for hidden layer	Sigmoid
	Activation function for output layer	Linear
	Training algorithm	IWO
	Cost function	Mean square error
IWO	Population size	50
	Variance reduction exponent	2
	Initial value of standard deviation	0.01
	Final value of standard deviation	0.001
	Maximum iteration	800

**Table 4:** Summary of influence of training set size on the prediction results. RMSE, root mean square error; MAE, mean absolute error.

Dataset	Size of Training Dataset (%)	Size of Testing Dataset (%)	R^2^	RMSE	MAE	ErrorStD	Slope
Training	10	90	0.987	0.029	0.025	0.030	0.974
	20	80	0.559	0.243	0.211	0.136	0.633
	30	70	0.897	0.081	0.066	0.067	0.984
	40	60	0.687	0.141	0.109	0.133	0.646
	50	50	0.821	0.115	0.100	0.114	0.883
	60	40	0.978	0.039	0.024	0.039	0.976
	70	30	0.952	0.061	0.047	0.061	1.011
	80	20	0.971	0.045	0.033	0.045	0.931
	90	10	0.977	0.043	0.031	0.042	0.952
Testing	10	90	0.387	0.226	0.144	0.205	0.535
	20	80	0.511	0.268	0.238	0.192	0.610
	30	70	0.737	0.151	0.108	0.138	0.890
	40	60	0.688	0.190	0.148	0.164	0.643
	50	50	0.826	0.125	0.106	0.124	0.860
	60	40	0.979	0.045	0.036	0.042	0.966
	70	30	0.982	0.041	0.029	0.041	0.961
	80	20	0.982	0.044	0.037	0.040	0.914
	90	10	0.987	0.038	0.029	0.040	0.914

**Table 5:** Performance indicators of the optimal FNN–IWO model.

Indicator	R^2^	RMSE	MAE	ErrorStD	Slope	Slope Angle
Training part	0.978	0.039	0.024	0.039	0.976	44.296°
Testing part	0.979	0.045	0.036	0.042	0.966	44.015°
All data	0.978	0.042	0.029	0.041	0.969	44.101°

**Table 6:** Error analysis of prediction performance with respect to different ranges of values of structural variables.

Structural Parameter	Lower Bound	Upper Bound	Number of Data	R^2^	RMSE (kN)	MAE (kN)
Depth/width ratio (-)	1	1.2	11	0.98	137.57	95.25
	1.2	1.4	22	0.98	71.07	56.01
	1.4	1.6	43	0.97	144.71	109.65
	1.6	1.8	11	0.89	56.16	38.91
	1.8	2	3	1.00	24.75	21.81
Thickness of steel tube (mm)	0	2	4	0.91	87.07	70.74
	2	4	52	0.91	118.58	80.36
	4	6	29	0.97	85.70	61.60
	6	8	8	0.91	178.54	143.84
	8	10	6	0.89	92.27	72.82
Yield stress of steel (MPa)	190	260	26	0.97	64.45	50.11
	260	320	6	0.97	146.68	99.40
	320	380	50	0.91	129.64	92.09
	380	440	8	0.99	137.34	104.47
	440	515	9	0.99	76.28	55.16
Compressive strength of concrete (MPa)	5	20	25	0.90	157.25	113.71
	20	30	22	0.93	120.57	84.45
	30	40	24	0.99	87.44	67.86
	40	50	28	0.99	75.40	53.80
Slenderness ratio (-)	0	20	78	0.98	123.29	86.64
	20	40	6	0.98	42.80	32.09
	40	60	13	0.99	72.25	55.32
	60	80	1	-	116.74	116.74
	80	100	1	-	49.65	49.65

**Table 7:** Comparison of performance indicators between FNN–IWO and individual FNN.

Data	Model Used	RMSE	MAE	ErrorStD	R^2^	Slope
Training	FNN–IWO	0.039	0.024	0.039	0.978	0.976
	FNN	0.087	0.070	0.075	0.923	0.893
	% Gain	+55.8	+65.9	+48.1	+6.0	+9.2
Testing	FNN–IWO	0.045	0.036	0.042	0.979	0.966
	FNN	0.087	0.071	0.071	0.919	0.952
	% Gain	+47.9	+49.2	+41.3	+6.5	+1.5
